# Construction Method of a Digital-Twin Simulation System for SCARA Robots Based on Modular Communication

**DOI:** 10.3390/s24227183

**Published:** 2024-11-09

**Authors:** Zihan Zhang, Qihui Guo, Maksim A. Grigorev, Ivan Kholodilin

**Affiliations:** Department of Electric Drive, Mechatronics and Electromechanics, South Ural State University, Chelyabinsk 454080, Russia; asp22ct750@susu.ru (Z.Z.); asp22gt944@susu.ru (Q.G.); grigorevma@susu.ru (M.A.G.)

**Keywords:** SCARA robot, digital twin, virtual-reality drive, robot motion simulation, robot operating system

## Abstract

Due to the high cost of robots, the algorithm testing cost for physical robots is high, and the construction of motion control programs is complex, with low operation fault tolerance. To address this issue, this paper proposes a low-cost, cross-platform SCARA robot digital-twin simulation system based on the concept of digital twins. This method establishes a 5D architecture based on the characteristics of different platforms, classifies data and integrates functions, and designs a data-processing layer for motion trajectory calculation and data storage for a virtual-reality robot. To address the complexity of data interaction under different cross-platform communication forms, an editable, modular, cross-platform communication system is constructed, and various control commands are encapsulated into simple programming statements for easy invocation. Experimental results showed that, based on modular communication control, users can accurately control data communication and synchronous motion between virtual and physical models using simple command statements, reducing the development cost of control algorithms. Meanwhile, the virtual-robot simulation system, as a data mapping of the real experimental platform, accurately simulated the physical robot’s operating state and spatial environment. The robot algorithms tested using the virtual simulation system can be successfully applied to real robot platforms, accurately reproducing the operating results of the virtual system.

## 1. Introduction

With the rapid development of digital technology and the rise of intelligent manufacturing [1], robots are widely used in industries [2], education [3], healthcare [4], aerospace [5], and services [6] and have become an indispensable part of modern industrial production [7]. This has driven the emergence of a series of automated processes, making modern industry more efficient, rapidly increasing productivity, saving significant amounts of materials and energy, and providing safer working conditions [8,9].

As one of the most commonly used robots in the industry, SCARA robots not only have complex control systems with multiple inputs and outputs but also exhibit time-varying and strongly coupled dynamic characteristics [10,11]. In integrated circuit wafer processing, SCARA robots, as an indispensable part, can transport and align wafers in different processes to meet the requirements of high speed and high precision in wafer handling [12]. Meanwhile, with the rapid development of 3D measurement technology, 3D vision sensors have been applied to robotic systems to perform object grasping tasks [13]. Wang et al. [14] built an experimental platform for the vision system based on the PointNetRGPE deep-learning method, combined with environmental factors, to estimate the grasping posture of SCARA robots, thereby improving the success rate of grasping. Chanal et al. [15] chose SCARA robots to describe and analyze their geometric behavior, improving inverse kinematics errors and enhancing geometric accuracy. To solve problems such as the position tracking of SCARA robots, Soriano et al. [16] designed and optimized a sliding mode controller. However, in real production, the complex and variable working environment poses a challenge for robots, while the tedious operational processes present another challenge for professional robot operators.

To address these issues, emerging enabling technologies such as digital twins [17,18,19] have emerged, allowing for the integration of physical equipment with virtual environments. The concept of the digital twin first appeared in Professor Grieves’ lecture on “Product Life-cycle Management”. Although the concept was initially vague, a 2014 paper [20] clarified that a digital twin (DT) includes physical products, virtual products, and the communication between them. NASA integrated multi-disciplinary and multi-scale simulation processes to map the real state of spacecraft in real-time, sparking increased discussion about digital twins [21]. Since then, digital twins have garnered increasing attention and research, with application demands continuously expanding and upgrading [22,23]. In Industry 4.0, Tao Fei proposed the concept and key technologies of the digital-twin workshop [24]. Building on the existing 3D digital-twin model, they introduced a five-dimensional (5D) digital-twin model, which included physical entities, virtual models, services, twin data, and connection threads [25].

However, while most digital-twin concepts share similar elements, Rodríguez et al. [26] propose that a key requirement for digital twins is the ability to self-update at regular intervals, with fault detection in variable conditions not triggering the digital-twin synchronization program. To achieve this, a pivotal step in this process is the detection and assessment of erroneous data, with fault detection technology maximizing operational availability and minimizing risks. Wang et al. [27] utilized patch variational autoencoding generative adversarial networks to propose an enhanced data generation model for rolling bearing fault diagnosis. Through layered sampling and stable structural design, they improved the network’s robustness and classification accuracy, effectively diagnosing and detecting faults.

With the widespread application of digital-twin technology, robotic digital twins have gradually become a research hotspot, attracting numerous scholars to study this topic. Du et al. [28] based their work on the framework of the digital-twin system, achieving real-time mapping between the physical and virtual layers and collecting and transmitting twin data to enable comprehensive real-time monitoring of robots in a virtual environment. Zhao et al. [29] constructed a design framework for a digital-twin industrial robot production line based on mechatronics and implemented visualization, simulation, and feedback control to promote the rapid practice of digital production lines. Zhang et al. [30] combined digital-twin technology with robotic CNC machines, establishing signal interaction, and designing an automated loading and unloading system for motion simulation, aiming to reduce costs, save time, and monitor signals. However, the focus of the aforementioned studies mainly remains on the virtual level, failing to achieve satisfactory interaction between physical entities and virtual models.

The challenge in digital-twin robotics research lies in driving the twin model’s motion through real-time data, thereby achieving virtual–real integration between physical and virtual models. Deep learning provides a critical means of coordinating virtual and physical systems, equipping robots with the capacity for efficient perception, precise analysis, and intelligent decision-making. Deep-learning models [31,32,33], based on neural network architecture, combine the ability to learn generalized feature patterns while capturing valuable information directly from data without manual feature extraction, leading to higher accuracy and sparking extensive research across various fields. The class-aware adversarial multiwavelet convolutional neural network (CAMCNN) introduces multiple wavelet convolution kernels to replace traditional convolution kernels and incorporates multiwavelet convolution layers with a class-aware mechanism. This network architecture serves as a feature extractor, efficiently capturing latent feature information to enable timely responses [34]. The trackable multi-domain collaborative generative adversarial network (TMCGAN) leverages multi-domain collaborative generation techniques, creating and simulating fault data across different operating conditions. This enables the classifier to perform intelligent fault diagnosis, even with imbalanced data, enhancing model transparency, boosting the credibility of generated data and diagnostic results, and improving model generalizability [35].

Matulis et al. [36] proposed a method for mapping learning from the virtual domain to physical-robot twins. Havard et al. [37] built a real-time co-simulation architecture between digital-twin and virtual-reality environments for workstation collaborative design and ergonomics research. Garg et al. [38] performed programming simulation for FANUC robots and established cross-platform communication between digital twins and physical robots (PR) to achieve robot trajectory motion and high-precision control. Yang et al. [39] proposed a digital-twin framework for flexible assembly robots, achieving bidirectional mapping between virtual and physical models and comprehensive monitoring of the assembly process. It can be seen that many scholars have achieved significant results in the operational functions and motion states of digital-twin robots.

However, in most studies, the constructed digital-twin systems have low user editability, insufficient data communication versatility in cross-platform simulations, and the virtual models constructed in most studies only target the robots themselves, lacking simulation of the robot operating environment and making it difficult to collect virtual environment information.

In view of this, to enhance both the editability and comprehensiveness of the simulation environment and the ease of control of the SCARA robot digital-twin system, this paper proposes a 5D construction method for a cross-platform, digital-twin system. This method builds the data-processing layer, virtual-robot layer, and physical-robot layer on different platforms according to the functional characteristics of each platform. A modular communication system is designed and integrated in the form of command statements to achieve bidirectional data acquisition and real-time communication control between different functional layers and communication forms. Finally, a virtual reality synchronization experiment test is conducted on the constructed digital-twin system to verify the mapping effect of the virtual and physical-robot systems and the accuracy of the simulation results.

## 2. The Structure of the SCARA Robot Digital-Twin Platform

**Figure 1:**
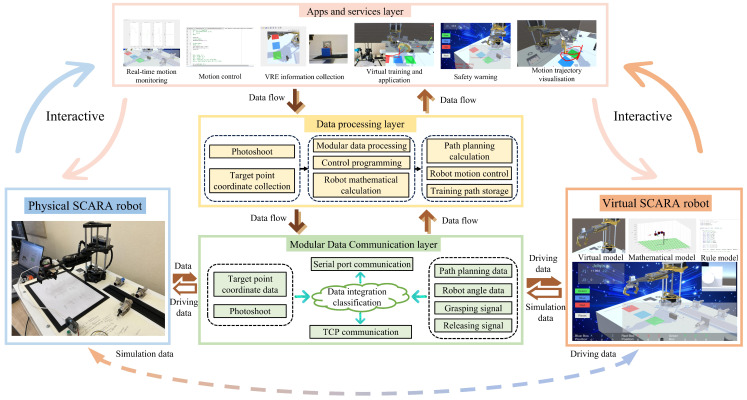
Platform structure diagram.

As a platform for data interaction and mutual mapping between virtual and physical robots, the digital-twin platform needs to achieve the free control and high-precision simulation of a virtual-reality robot while meeting the requirements of 3D visualization monitoring [40]. In view of this, based on the digital-twin architecture proposed by Fei Tao [41], this paper designs a multi-platform cooperative digital-twin system architecture for SCARA robots, as shown in Figure 1.

The entire system structure is divided into five parts: the physical-robot layer, the virtual-robot layer, the data-processing layer, the modular data communication layer, and the apps and services layer. The physical-robot layer is the experimental platform for the real SCARA robot, providing data support for the construction of the virtual robot and receiving data and control commands from the virtual layer. The virtual-robot layer, as the twin mirror of the real environment, is combined with the data-processing layer to handle the virtual motion debugging of the SCARA robot and visualize the operating environment and motion parameters of the real SCARA robot. In the data-processing layer, the operational data of the virtual-reality robot will be collected, the robot’s motion trajectory will be calculated, and the execution of robot actions will be controlled. The modular data communication layer serves as the data bridge between the layers of the digital-twin system, providing bidirectional communication data transmission for various functional platforms, thereby integrating different functional platforms to achieve joint simulation. In the apps and services layer, a visual interactive interface is constructed to facilitate users in viewing various data of the virtual-reality robot. Additionally, the robot control system is encapsulated into code statements to simplify the user’s simulation control operations.

## 3. Construction of Virtual-Reality SCARA Robot Experimental Platform

**Figure 2:**
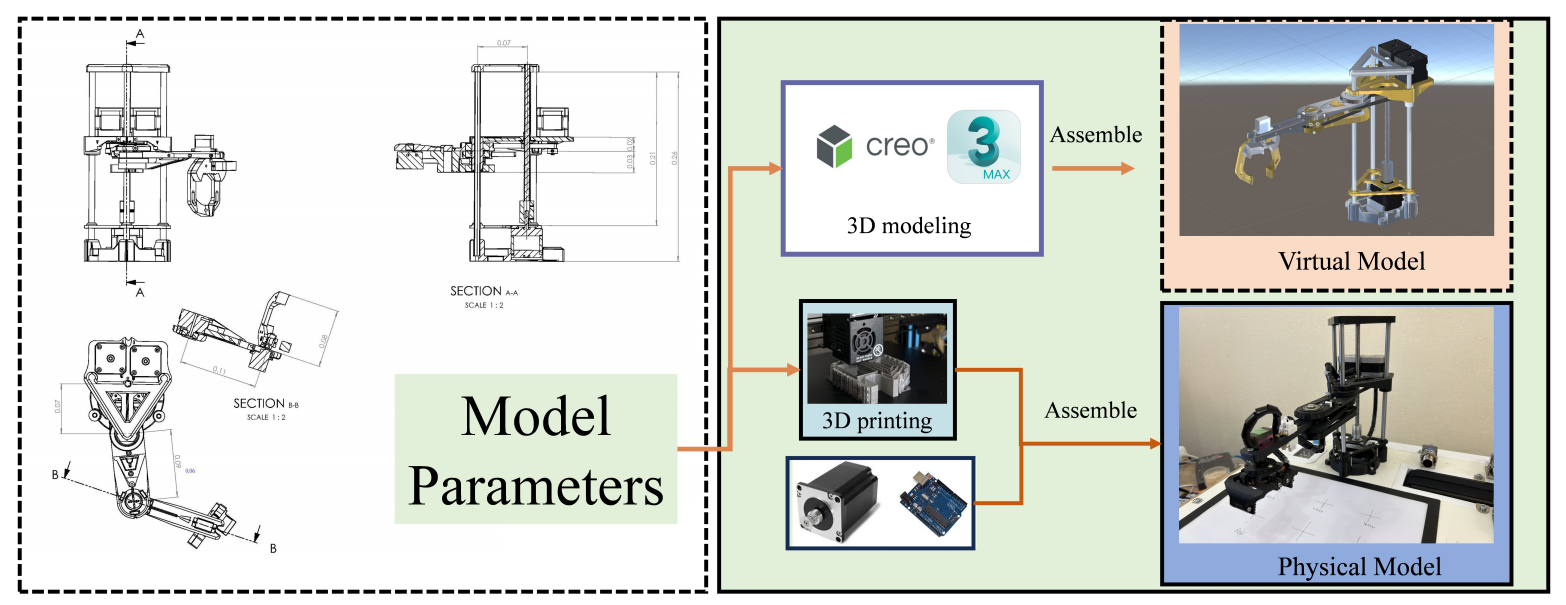
SCARA robot structure diagram.

The robot used in this research is a SCARA robot arm, which has four degrees of freedom, including rotational freedom around the X, Y, and Z axes and translational freedom along the Z axis. Its dimensional parameters are shown in Figure 2. According to the design drawings, each joint arm of the robot was manufactured using a 3D printing platform, and the movement of each joint was driven by a servo motor. After the robot was assembled, an Arduino control board was added to it, enabling computer control of the robot through USB serial communication. Due to the limitations of the Unity3D platform’s modeling precision, the virtual model of the SCARA robot was constructed in Creo, then imported into 3Dmax to add materials and enhance its appearance. Finally, the complete model was imported into Unity3D, and connection and interaction were implemented through C# program scripts.

**Figure 3:**
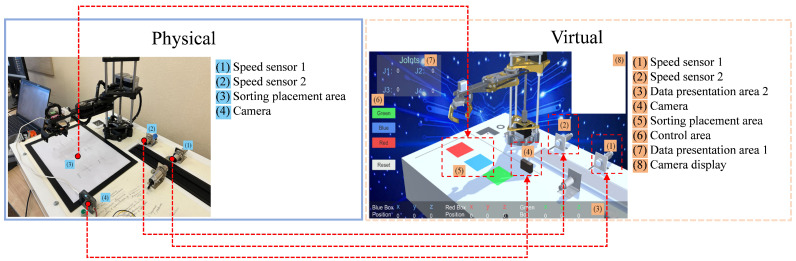
Distribution diagram of the robot physical platform and virtual system structure.

In the physical experimental platform, besides the SCARA robot, there were also speed sensors, camera, and sorting placement area, as shown in Figure 3. Similarly, in the virtual system, corresponding models for speed sensors, camera, and sorting placement areas were constructed. Additionally, to enhance the visualization of robot data in the digital-twin system and simplify the user’s operation of the system, a control UI was constructed in the virtual system. The UI control interface area was divided into three parts: Data presentation area 1, Control area, and Data presentation area 2. Data presentation area 1 showed real-time data of the robot joints. The control area consisted of three colored buttons and a reset button. The colored buttons were used to generate virtual cubes of corresponding colors, while the reset button deleted all cubes from the interface. Data presentation area 2 showed the real-time position information of each colored cube.

## 4. Design of the Modular Data Communication System

### 4.1. Data Reception and Transmission

**Figure 4:**
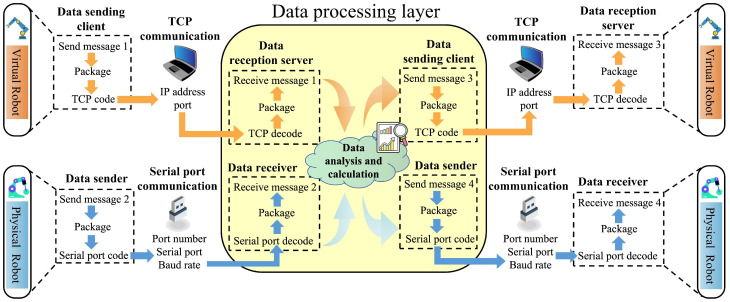
Cross-platform data transmission and reception architecture.

In multi-platform concurrent operations, due to the different underlying logic codes of each platform, data cannot be directly called between platforms. To ensure data interaction between multiple platforms within the digital-twin system, a cross-platform data transmission system needs to be constructed. The data transmission system designed for this platform is based on a TCP transmission protocol and a serial port communication protocol. The TCP transmission protocol is used for communication between the virtual-robot layer and the data-processing layer, while the serial port communication protocol is used for communication between the physical-robot layer and the data-processing layer [42]. The data-processing layer serves as the interaction center for cross-system data, integrating data from different platforms and controlling the data flow. The architecture of the platform communication system is shown in Figure 4.

The TCP protocol is a transport layer communication protocol based on byte streams [43]. During transmission, it segments large data packets and manages them as packets of message segments. Communication based on the TCP protocol requires the construction of a data reception server and a data sending client in both the virtual-robot layer and the data-processing layer. Under this communication architecture, the data collected by the virtual robot is loaded into the virtual simulation layer client as data packets. The client establishes a network connection using the TCP/IP protocol and transmits the data to the specified target server. The input parameters for this transmission method are only the IP address and port number of the target device, and the data to be transmitted. Before sending data, the input data will be encoded, converting the original data type into a byte array in UTF8 encoding form and calculating the size of the data. The data size and message data are then written to the specified TCP connection. Similarly, on the server side, after receiving the specified information at the IP port, the information is converted into a string using UTF8 decoding. The restored information packets are then classified, and the data are converted to double-precision format to facilitate subsequent classification calculations by the system.

The stepper motors of the physical robot are controlled by an Arduino development board, which uses serial port communication via the USB port to achieve low-cost device control. In serial port communication, the devices are connected to the computer via physical data signal lines and data is transmitted to the specified device based on the COM port number. Unlike TCP communication, in Arduino serial data communication data is written to the port in string format. When the data-processing layer reads port data, the decoding system needs to convert the string data to double-precision format. Conversely, when writing data to the port, the encoding system needs to convert the double-precision data to string format. This facilitates the interactive analysis of sensory and simulation data, providing a basis for further data computation.

### 4.2. Transmission Data Integration and Classification

**Figure 5:**
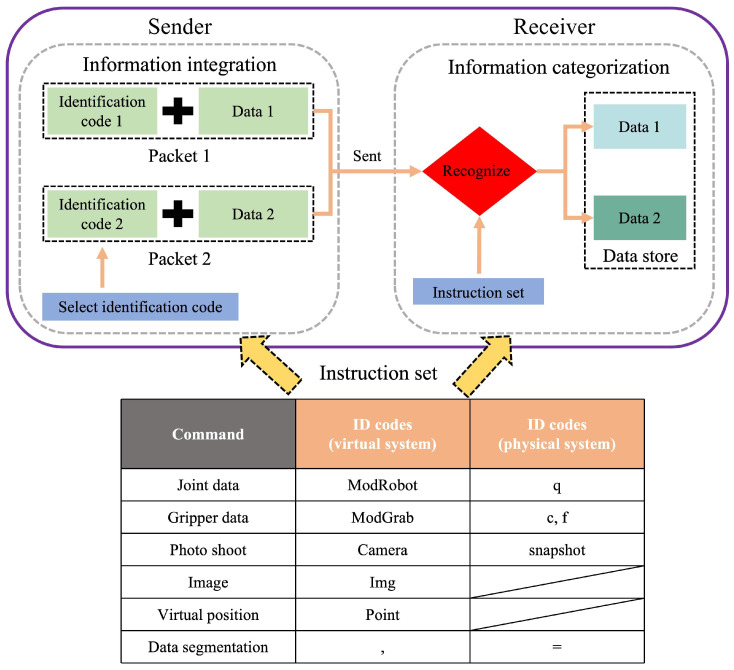
Flowchart of information integration and identification.

During the entire motion process of the virtual-reality robotic arm, five types of data transmission are mainly required: robotic arm joint data, gripper grasp and release commands, camera photo shoot commands, image data, and virtual position information. For these five types of data, this paper designs a data packet integration function at the data sender and a data packet categorization function at the data receiver to achieve rapid classification of data in each functional layer. The operating principle is shown in Figure 5. At the sender, the data integration system inserts a data type identification code at the beginning of the data group according to the command type, storing the data type information at the first position of the string and the specific data information after the data type information. At the receiver, after receiving the transmitted data, the classification system determines the data type based on the initial characters and extracts the specific data. The data information is then categorized into different arrays for storage. By introducing a command identification code, the data at the sender does not need to follow a fixed data arrangement order. The receiver can only store the corresponding data when the category identification code is recognized. This avoids interference from irrelevant information during data transmission and allows for the independent transmission of single-type data packets as well as the mixed transmission of multiple types of data.

Furthermore, during data transmission, the single-type information sent is usually composed of multiple sets of strings. To enable the system to better distinguish these pieces of information, the model uses delimiter characters for data separation. The delimiter can split the string groups into substrings, facilitating data classification and identification. Therefore, the data transmission formats are as shown in Equations (1) and (2).

(1) $${TD}_{V}=\left\{IS,DataA,DataB,\ldots \right\}$$

(2) $$TD_{R}=\left\{IS=DataA=DataB=\ldots \right\}$$

where $TD_{V}$ represents the transmission data of the virtual layer, $TD_{R}$ represents the transmission data of the physical layer, IS is the identification code, and DataA and DataB are the operational data for the virtual and physical layers.

### 4.3. Construction of the Data Transmission Module

**Figure 6:**
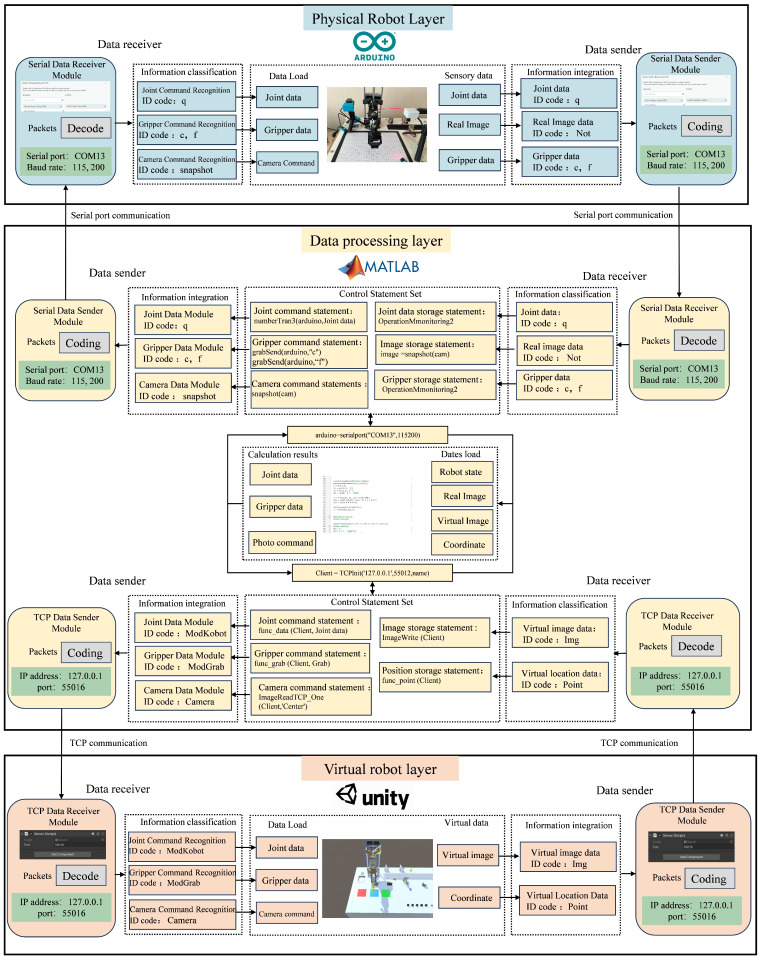
Cross-platform data transfer module build diagram.

In this digital-twin system, the virtual-robot layer is built on the Unity3D platform, the physical-robot control system is constructed on the Arduino platform, and the data-processing layer is implemented on the MATLAB R2022a platform. Based on the proposed data communication system architecture, the specific construction of data receivers and senders is carried out on these three platforms. The construction structure is shown in Figure 6.

In the virtual layer, due to the introduction of identification codes, the virtual-robot platform can use the same IP address and port number to create clients and servers. The decoding and coding functions are integrated into the data transmission ports and encapsulated using C# programming, reducing the number of modules for easier platform invocation. The TCP communication module script in the virtual layer can customize the IP address and port number according to user requirements to accommodate different experimental devices. For this TCP system, the IP address used is ‘127.0.0.1’, and the port number is ‘55016’. Based on the configured TCP communication address, the data-processing layer connects to the virtual layer using the MATLAB built-in TCP Init function. In the physical layer, the physical robot connects to the computer via a USB interface, and the serial connection is completed in Arduino based on the serial port number and the development board model. Similar to the virtual layer port settings, the physical layer serial port address can also be changed according to user requirements. The decoding and coding functions are integrated and encapsulated using C++ programming. In the data-processing layer, the serial connection is established using MATLAB’s built-in serial port function.

According to the information integration and identification system designed in Figure 5, the combination of command information is identification code (initial character) + operational information. Therefore, corresponding information categorization and integration modules are constructed in both the virtual and physical layers to complete command recognition and operational data feedback. As the central control of the digital-twin system, the data-processing layer is responsible for sending control commands and detecting robot operational data. To simplify control and monitoring operations, the communication addresses and command identification codes are modularly encapsulated according to control commands. This means that the information integration and classification modules in the data-processing layer are separately encapsulated in modular statements. Users only need to call the encapsulated control-statement set in the data-processing layer and input the control data to complete the corresponding command, thereby simplifying the control operations of the virtual and physical robots.

Compared to traditional control systems, the communication module designed in this paper simplifies the robot simulation testing process. Users only need to call the control command set encapsulated in the data-processing layer, input control data, and execute the test algorithm to complete the simulation testing of the robot’s functions, effectively reducing control commands to a single instruction. This design abstracts complexities such as underlying communication and multi-node dependencies. Users are not required to learn data synchronization, format conversion, or fault tolerance mechanisms and can focus solely on optimizing their algorithms, thereby simplifying the implementation process of control algorithms and making the construction and debugging of control systems more efficient and convenient.

## 5. Virtual-Reality Synchronous Operation of SCARA Robots

### 5.1. Digital Mirror and Monitoring

In the digital-twin system, ’digital mirror for reflection’ refers to the real-time detection of the physical model’s operational state by the virtual model, with physical data being loaded into the virtual model. Utilizing the advantages of 3D virtual display, it more intuitively shows the operating parameters and trajectories of the physical model, overcoming the limitations of monitoring the physical model in time and space. According to the designed digital-twin system architecture, this section will describe the construction and operation methods of digital mirroring for the SCARA robot.

**Figure 7:**
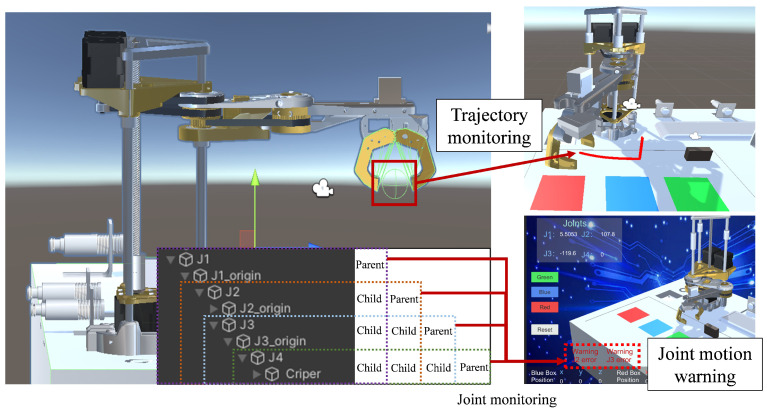
Virtual robot joint relationship settings and data monitoring.

In the physical robot, the joint data are directly loaded into the motors at each joint position using physical control buttons, thereby enabling the model’s movement. Since the joint data are sent one by one to the Arduino control board, in order to make the joints of the robot arm run at the same time an array of joints is created in Arduino to store the transmitted joint data. Once data reception is complete, the control board simultaneously loads the data into the corresponding motors, allowing all joints to operate simultaneously. In the virtual-robot model, there are no physical constraints between the joints. To achieve coordinated follow-up motion of the robot arm, it is necessary to set up parent–child relationships based on coordinates in Unity3D for each joint model. This enables the joint models to form a nested structure, where in the local coordinate system, when the parent node rotates or moves the child joints follow the motion based on the parent joint’s origin coordinates. The parent–child structure of each joint in this model is shown in Figure 7. Joints J2 to J4 are nested within the previous joint, and joint J1 is the parent node of all joints. Additionally, to better monitor the operation of the physical and virtual models, a joint motion warning module and a trajectory tracking module are added to the virtual-robot joints. These modules monitor the set joint data and the robot’s end position, displaying the real-time monitoring results on the UI interface to enhance system operation safety.

**Figure 8:**
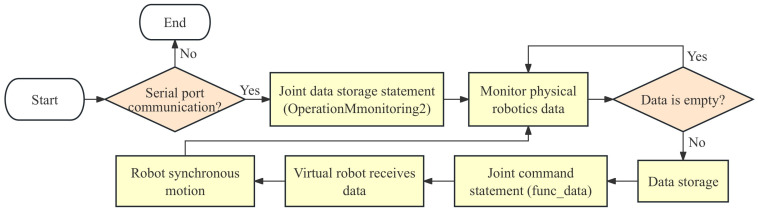
Flowchart of digital mirror and monitoring.

In the design of a modular data communication system, this paper encapsulates a set of control statements in the data-processing layer. Among them, the ‘OperationMonitoring2’ statement serves as a data storage function for the joint operation data of the physical robot. This function reads the motor data of each joint of the physical robot in real-time through a configured serial port address. The data-processing layer converts the motor data into operating angles and stores it in real-time. After collecting the operation information of the physical robot, the virtual-robot joint command statement ‘func_data’ in the control-statement set is called, and the collected physical joint data is inserted into the statement. Then, TCP communication is used to send the data to the Unity3D. Based on the joint data of the physical model sent by the data-processing layer, the server in Unity3D parses the data set into joint-angle data according to the identification code of the data origin. Using the ‘transform.localRotation’ function in Unity3D, the data are synchronously loaded into each joint, allowing the virtual joints to rotate or move accordingly. This completes the real-time mapping of the virtual model to the physical model, as shown in the workflow diagram in Figure 8.

### 5.2. Digital Control

**Figure 9:**
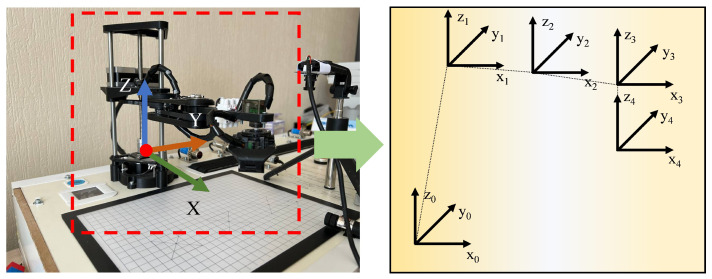
SCARA robot structure and D–H coordinate system.

In the digital mirroring system, the virtual model needs to load the actions of the physical model in real-time, and the control of the physical model still requires manual operation. However, in the digital control system the digital-twin system needs to calculate the robot’s joint movements and operating trajectories based on the virtual model’s instructions and use control commands to transmit the results to the physical robot, achieving operational control from the virtual layer to the physical layer. This section describes the data communication control from the virtual layer to the physical layer and the implementation of robot trajectory planning. In this system, the D–H (Denavit–Hartenberg) method is used to describe joint J1 of the SCARA robot as a prismatic joint, while J2, J3, and J4 are described as revolute joints. The results of the joint descriptions are shown in Table 1, and the distribution of the link structures and coordinate systems is illustrated in Figure 9.

Based on the standard D–H coordinate system constructed, the forward kinematic analysis of the SCARA robot is conducted. The transformation matrix between the two links is known to be:

(3) Tii−1=cosθi−sinθicosαisinθisinαiaicosθisinθicosθicosαi−cosθisinαiaisinθi0sinαicosαidi0001

By multiplying the transformation matrices between the links, it is possible to derive the homogeneous transformation matrix for the SCARA robot from the base coordinate system to the end-effector coordinate system as:

(4) T40=T10T21T32T43=R40p400001

where R40 represents the rotation matrix of the robot’s end-effector in the base coordinate system, indicating the end-effector’s pose, and p40 represents the spatial coordinates of the robot’s end-effector in the base coordinate system, indicating the end-effector’s position.

In the inverse kinematic calculations of the SCARA robot, due to the relatively simple structure of the SCARA robot, the system employs the geometric method to solve the inverse kinematics. In the SCARA robot, movements in the X and Y directions are controlled by the rotation of links L2 and L3, while movements in the Z direction are controlled by the movement of link L1. Given the known end-effector coordinates, the movement of link L1 can be directly determined:

(5) $$d_{1}=z-d_{2}-d_{3}-d_{4}$$

where *z* represents the Z-axis value of the end-effector coordinates and $d_{2}$, $d_{3}$, and $d_{4}$ are the link offsets for links L2, L3, and L4, respectively.

**Figure 10:**
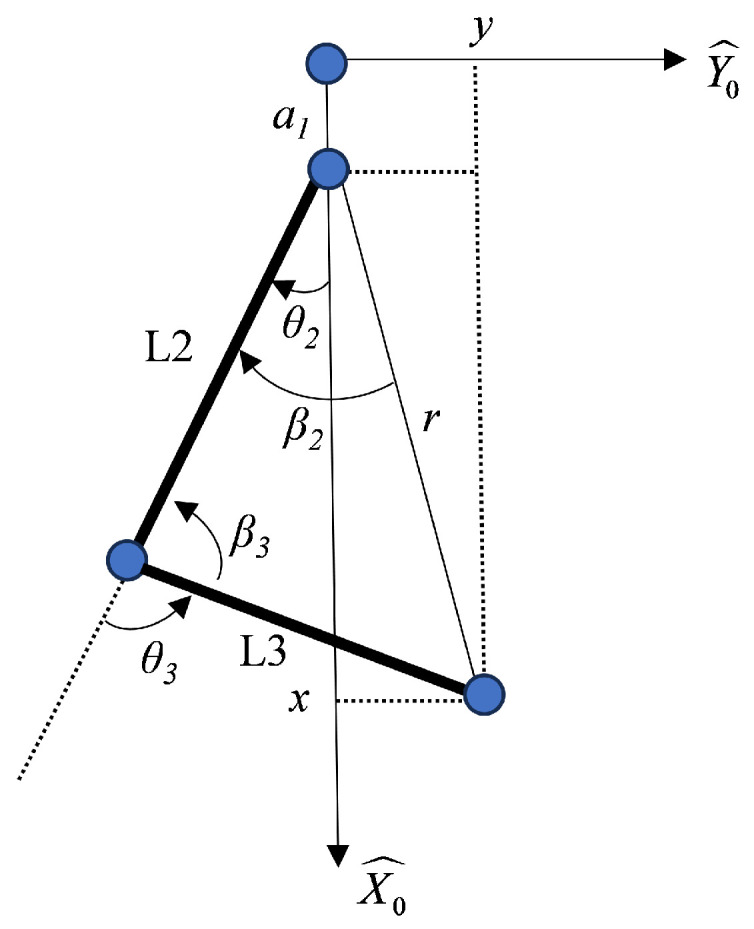
SCARA robot motion plane diagram.

The motion plane diagram of the SCARA robot’s L2 and L3 links is shown in Figure 10, where links L2, L3, and the end-effector coordinates form a triangle. The two sides of the triangle are the lengths of links L2 and L3, respectively. By determining the length of the third side, *r*, all internal angles of the triangle can be calculated using the law of cosines. From the planar diagram, *r* is the line connecting the end-effector coordinates and the J2 node, and its solution formula is:

(6) $$r={\left(x-a_{1}\right)}^{2}+y^{2}$$

where *y* represents the Y-axis coordinate of the robot’s end-effector, $a_{1}$ represents the link length of link L1, and *x* is the X-axis coordinate of the robot’s end-effector.

After solving for *r*, the angle $\theta_{3}$ of L3 can be calculated using the law of cosines.

(7) $$\theta_{3}=180^{\circ }\pm arccos((a_{2}^{2}+a_{3}^{2}-r^{2})/\left(2\cdot a_{2}\cdot a_{3}\right))$$

where $a_{2}$ and $a_{3}$ represent the link lengths of L2 and L3, respectively.

Due to structural limitations, the motion angle of link L3 cannot exceed 180 degrees. Therefore, it is necessary to transform Formula (7) to convert angle values greater than 180 degrees into reverse motion, as follows:

(8) $$\theta_{3}=\pm \left(180^{\circ }-arccos\left(\left(a_{2}^{2}+a_{3}^{2}-r^{2}\right)/\left(2\cdot a_{2}\cdot a_{3}\right)\right)\right)$$

It can be observed that when the sum of the lengths of L2 and L3 is less than *r*, two sets of solutions for $theta_{3}$ can be derived. This indicates that there are two ways for the robot to rotate to the same point. The solutions for $theta_{2}$ are divided into two types based on the values of $theta_{3}$. When $theta_{3}$ is less than 0 degrees:

(9) $$\theta_{2}=\arctan\left(y/\left(x-d_{1}\right)\right)+\arccos\left(\left(a_{2}^{2}+r^{2}-a_{3}^{2}\right)/\left(2\cdot a_{2}\cdot r\right)\right)$$

When $\theta_{3}$ is greater than 0 degrees:

(10) $$\theta_{2}=\arctan\left(y/\left(x-d_{1}\right)\right)-\arccos\left(\left(a_{2}^{2}+r^{2}-a_{3}^{2}\right)/\left(2\cdot a_{2}\cdot r\right)\right)$$

Given the existence of two solutions, the robot must choose one for implementation. Here, the solution with the minimal joint motion, which is the nearest solution, is selected for loading. After calculating $\theta_{3}$ and $\theta_{2}$, the rotation matrix of L4 can be derived from the given end-effector pose matrix, and subsequently the rotation angle of L4 can be obtained:

(11) R43=R30R4−10

where R43 represents the rotation matrix of link L4 and R30 represents the rotation matrix of the end of link L3 relative to the base coordinate system.

**Figure 11:**
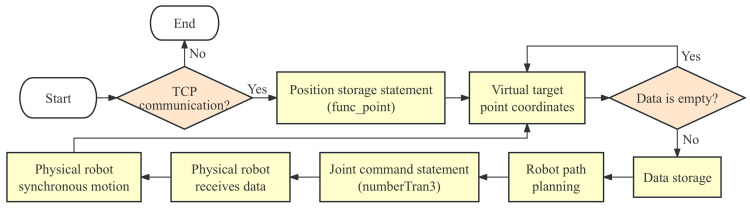
Flowchart of digital control.

After calculating all the joint motion data of the robot at the target point coordinates, path planning between the starting point and the coordinate point is required. Here, the Robotic Toolbox in MATLAB is utilized. Based on the joint data of the starting point and the calculated joint data of the target point, the toolbox uses interpolation algorithms to compute the interpolation of each joint angle and generates corresponding matrices to store the joint motion time series. In the constructed set of control statements, the position storage statement ‘func_point’ can transmit the position coordinates of the object to be grabbed in the virtual environment to the data-processing layer. Based on the coordinate position, the data-processing layer calculates the end-effector pose matrix at the target position of the robot. Through the aforementioned inverse kinematics calculations and path planning, the operation data of each robot joint are stored. At this point, the command statement ‘numberTran3’ in the control statement set for the physical robot joints is called. This statement inserts the stored joint data into the command statement, which then writes the data to the Arduino via serial communication, thereby controlling the movement of the physical robot. This achieves the control of the physical model by the virtual model, and its working logic is shown in Figure 11.

### 5.3. Digital Prediction and Interaction

Utilizing virtual models to replace physical models for robot motion simulation and prediction in a digital-twin system is significant. The introduction of virtual models reduces the cost of training computations and addresses the safety hazards and potential damage associated with testing physical models. This section describes the interaction principles between the virtual-robot model and the virtual target. Additionally, a virtual camera is incorporated to capture images of the virtual environment. Combined with the control-statement set, this enables virtual simulation training and result storage.

**Figure 12:**
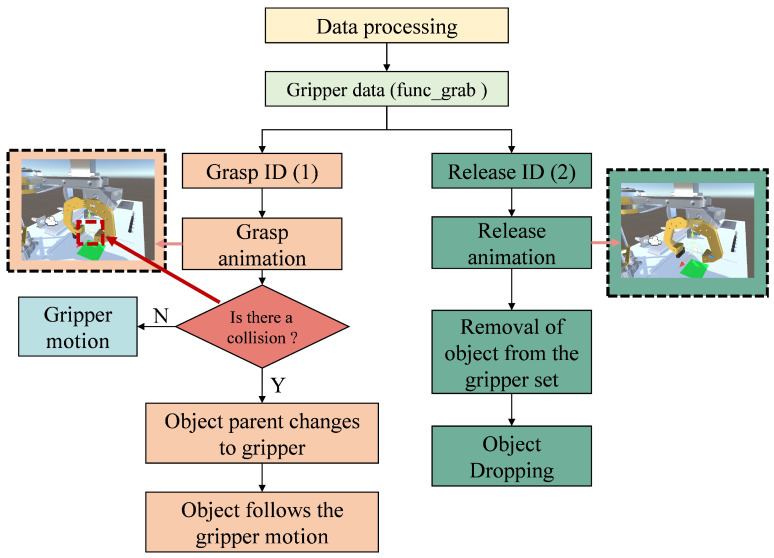
Operational logic diagram of the gripper’s actions.

The motion simulation of the gripper is different from that of the joints. In this paper, animation effects for the gripper’s grasping and releasing are created using the Animator component in Unity3D. When Unity3D receives a grasping or releasing command from the data-processing layer, the model will play the corresponding animation effects. In the gripper model, a transparent rigid body model is set below it to detect whether or not there is an object beneath the gripper. Figure 12 shows the operational logic diagram of the gripper’s actions. It can be seen that in the grasping state, when there is an object beneath the gripper, the program will detect a collision with the rigid body and the virtual object will be placed under the gripper, becoming its child object. At this point, the position and rotation of the virtual object will remain relatively consistent with the gripper. In the releasing state, the program will set the parent of the virtual object to null, removing it from the gripper’s child objects. It will also set the ‘isKinematic’ property of the virtual object to false, making it non-static. In this way, the virtual object will be affected by the physics engine, can be acted upon by external forces, and its position and rotation will be updated according to the physics engine’s calculations.

**Figure 13:**
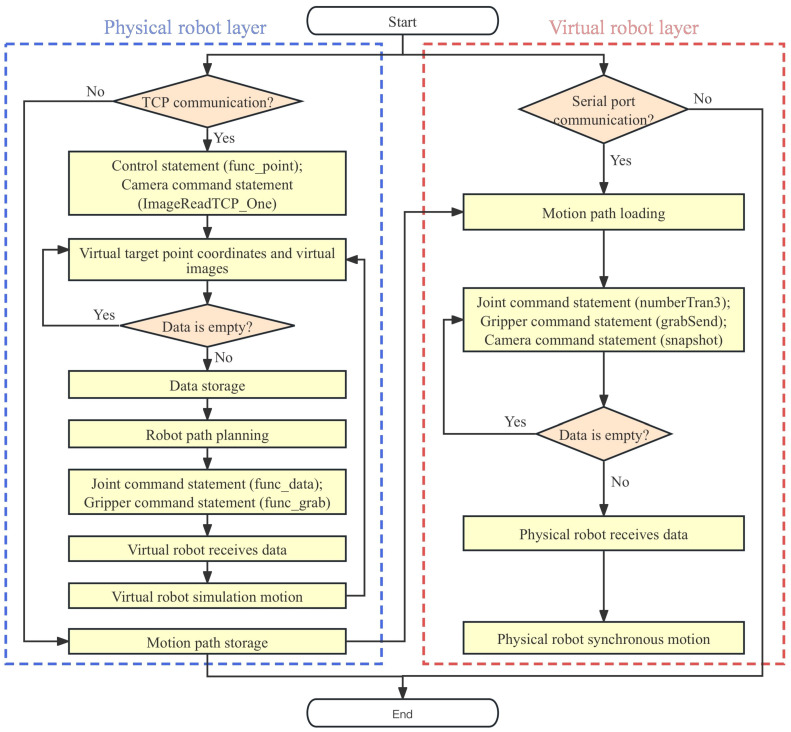
Training logic diagram of digital prediction and interaction.

In the virtual layer, apart from the main camera used to display the system’s main interface, an additional observation camera is set up to capture image information of the virtual environment. When the data-processing layer sends a command to take a photo, the camera system reads the pixel data of the virtual environment and creates a temporary Texture2D object, applies the rendering result to this texture, and then converts the texture into a byte array in PNG format. Via the TCP protocol, the byte array is sent to the data-processing layer, where a file named ‘image.png’ is created, and the data are written to this file in uint8 format, completing the image transmission. Based on the constructed virtual-robot interaction system, the encapsulated set of control statements in the data-processing layer can be used to manipulate the virtual robot for tasks such as virtual environment image capture, object grasping, and joint trajectory calculation, thus completing robot virtual simulation and algorithm training. Combined with the control statements for the physical robot, the results of the virtual simulation can be loaded into the physical robot after the virtual simulation is completed, thereby achieving algorithm testing and practical application, and its working logic is shown in Figure 13.

## 6. Experiment and Analysis

To validate the feasibility and reliability of the robotic digital-twin system under a modular communication architecture, performance testing of the constructed system is necessary. Based on the digital-twin maturity level requirements proposed by Tao Fei [17,25,44], this test experiment is divided into two parts: (1) Synchronization testing between the virtual robot and the physical robot, to validate the effectiveness of the virtual mapping and control entities in the system and the real-time performance of the data transmission system; (2) Simulation and validation testing of the virtual robot, to assess the effectiveness of the virtual model’s predictive simulation of the physical entity within the constructed system. The CPU core processor of the computer used in this experiment is an AMD 5600U, and the graphics card is an NVIDIA MX450.

### 6.1. Robot Virtual-Reality Synchronization Experiment

In the synchronization experiment of the robotic digital-twin system, the movement of the physical robot is initially controlled using physical control buttons. Then, a set of control commands is used to link the virtual model to verify the mapping accuracy of the virtual layer to the physical layer’s movements. Next, the robot’s grasping path is planned according to the coordinates of the target object in the virtual layer, and this planned path is loaded into the physical robot using command sets to verify the accuracy of the data loading. In the performance testing of the robot, the comparison of joint angles serves as an important metric for assessing performance error [38,45]. Therefore, this experiment evaluates whether or not the digital-twin requirements are met by comparing the joint-angle values of the physical robot with those of the virtual robot during each movement.

**Figure 14:**
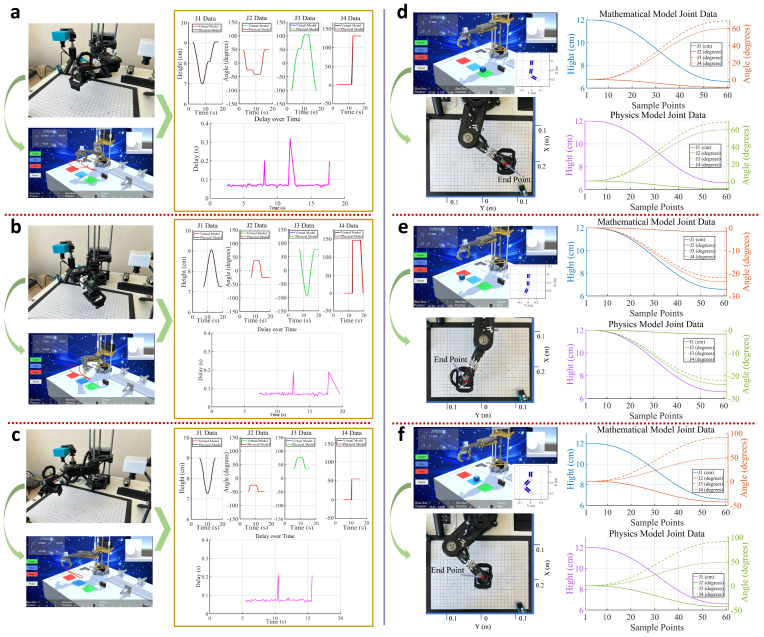
Virtual reality synchronization experiment results. (**a**–**c**) Three groups of experiments on the effect of the virtual robot on the operation state mapping of the physical robot. (**d**–**f**) Three groups of experiments in which the virtual robot controls the operation of the physical robot.

Three motion experiments were conducted on the physical robot using physical control buttons. The experimental results are shown in Figure 14a–c, which include actual running images of the virtual and physical models, joint-angle graphs monitored by the data-processing layer, and data reception delay graphs. From the joint-angle graphs, it can be seen that the virtual robot successfully tracked the physical robot’s movements under three different running trajectories. During the operation, the joint angles of the virtual and physical robots coincided, with no data loss or sudden changes, indicating that the data transmission system could accurately send and receive various parameters. During the experiments, the data transmission time from the physical robot to the virtual robot was measured, which was used to construct a delay graph for virtual-robot data reception. It can be observed that, during the three experiments, delay spikes occurred only at individual monitoring points, while the overall delay remained stable. The average delays for the three experiments were 0.0744 s, 0.0725 s, and 0.0764 s, respectively, meeting the synchronization requirements of the robot digital-twin system. Additionally, as a monitoring terminal, the virtual system can display the joint angles of the robot in real-time and draw the robot’s 3D motion trajectory based on the physical robot’s motion data, facilitating users to observe the physical robot’s operating status.

In the digital control experiment, a target object was first generated in the virtual system using the UI interface, and its coordinates were set. These virtual coordinates were transmitted to the data-processing layer via control statements. The data-processing layer planned the robot’s motion trajectory based on the coordinates, calculated the joint motion data for 60 sampling points, and loaded them into the physical robot using control statements, enabling the movement from the initial point to the target point. Figure 14d–f show the results of the three virtual-to-physical control experiments, where different virtual coordinates were set for the target object in each experiment. By comparing the joint data of the mathematical model and the physical model, it can be seen that the physical model’s trajectory was basically consistent with the data-processing layer’s calculated results, indicating that the data transmission system accurately loaded the calculated results into the physical model. To observe the operating status of the physical robot more intuitively, the posture at the robot’s end sampling point was collected and compared with the calculated model’s posture. The end posture of the physical robot was consistent with the calculated results, and the end-effector coordinates of the physical robot in the three experiments were (0.2071 m, 0.0662 m, −0.0199 m), (0.2422 m, −0.0421 m, −0.0195 m), and (0.1952 m, 0.0076 m, −0.0197 m), respectively. The absolute errors from the corresponding virtual coordinates were 2.01 mm, 3.83 mm, and 2.94 mm, indicating that the accuracy met the requirements for teaching simulation.

To further validate the advantages of the improved digital-twin data transmission system we proposed, comparative experiments were conducted on two main components: the virtual mapping entity and the virtual control entity. The data transmission framework used for comparison in the system is the traditional single-class data transmission mode [46], which was applied in the initial development phase of this system. For the virtual-robot mapping to the physical robot, five sets of action commands were programmed for the physical robot. Under the same action command, data transmission was conducted using both data transmission systems, and the average mapping delay rate of the virtual model to the physical model was recorded for each experimental set. Similarly, in the virtual robot controlling the physical-robot component, five different target points were set in the virtual environment. Under the same target point, the physical robot’s operation was controlled using both data transmission systems, with the run time to reach the target point recorded for each system.

**Figure 15:**
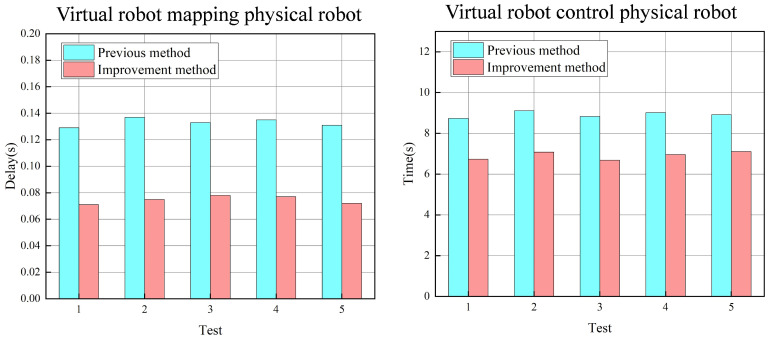
Comparison of the operation effects of the traditional data transmission architecture and the improved data transmission system in this paper.

The test results are shown in Figure 15. In the virtual mapping entity component, the traditional communication system, as a point-to-point data transmission mode, transmits only one data type at a time. Consequently, the physical model must send operational parameters individually, and the virtual model, upon receiving operational data from the physical model, must likewise identify and load each data point one by one. In the five test experiments, the average mapping delay with the improved communication system was significantly lower than that of the traditional method, reducing the average delay by 0.05 s compared to the traditional communication system. In the virtual control entity experiment, although both systems used the same trajectory calculation method, the improved communication system’s data integration module allowed the robot joint data calculated by the data-processing layer to be sent directly to the control sensors of the physical robot, eliminating the need for additional data filtering and classification by the physical sensors. Thus, compared to traditional communication methods, the improved system reduced the control response time from the virtual model to the physical model, thereby increasing the operational speed of the physical robot. This enables the physical robot to complete the same specified actions more quickly under the improved system, further enhancing the real-time control between the virtual and physical systems.

### 6.2. Virtual-Robot Simulation and Verification Experiment

**Figure 16:**
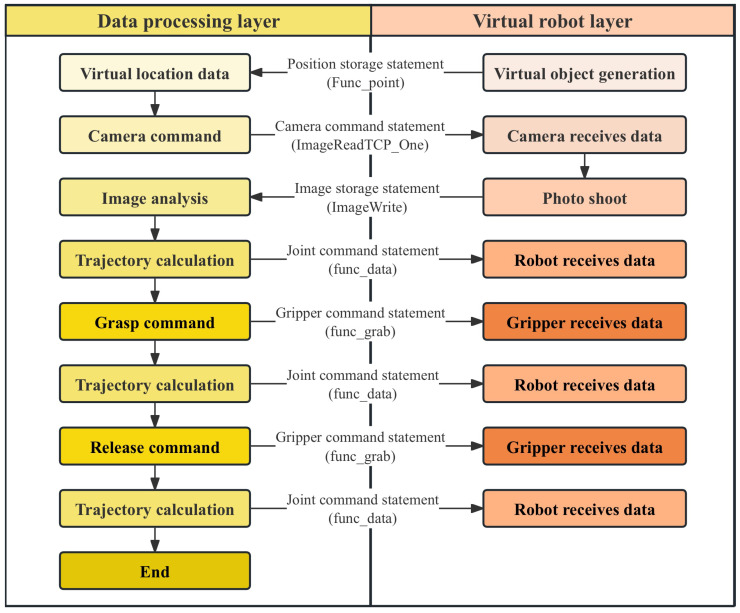
Operational logic of robot sorting simulation experiment.

To verify the accuracy of the virtual simulation system’s mapping to the real experimental platform, object sorting simulations were conducted on the virtual-robot platform. The final simulation results were then loaded onto the physical-robot platform to apply the simulation results. This experiment mainly simulated the robot arm’s recognition and classification of objects of different colors. In the experiment, blue, red, and green cubes were generated in the virtual system using the cube generation button on the user interface (UI). Subsequently, the data-processing layer performed robot trajectory planning calculations based on the positions of target objects in the virtual space and analyzed the images taken by the virtual camera, enabling the robot to perform color recognition and grasping tasks. The algorithm’s operational logic is shown in Figure 16.

To complete the classification of cube colors, an image color recognition program needs to be written in the data-processing layer. When the data-processing layer receives virtual images from the virtual simulation system, the program extracts the red, green, and blue channels of the images and assign them to the variables R, G, and B, respectively. The system determines the primary color by comparing the values of R, G, and B.

**Figure 17:**
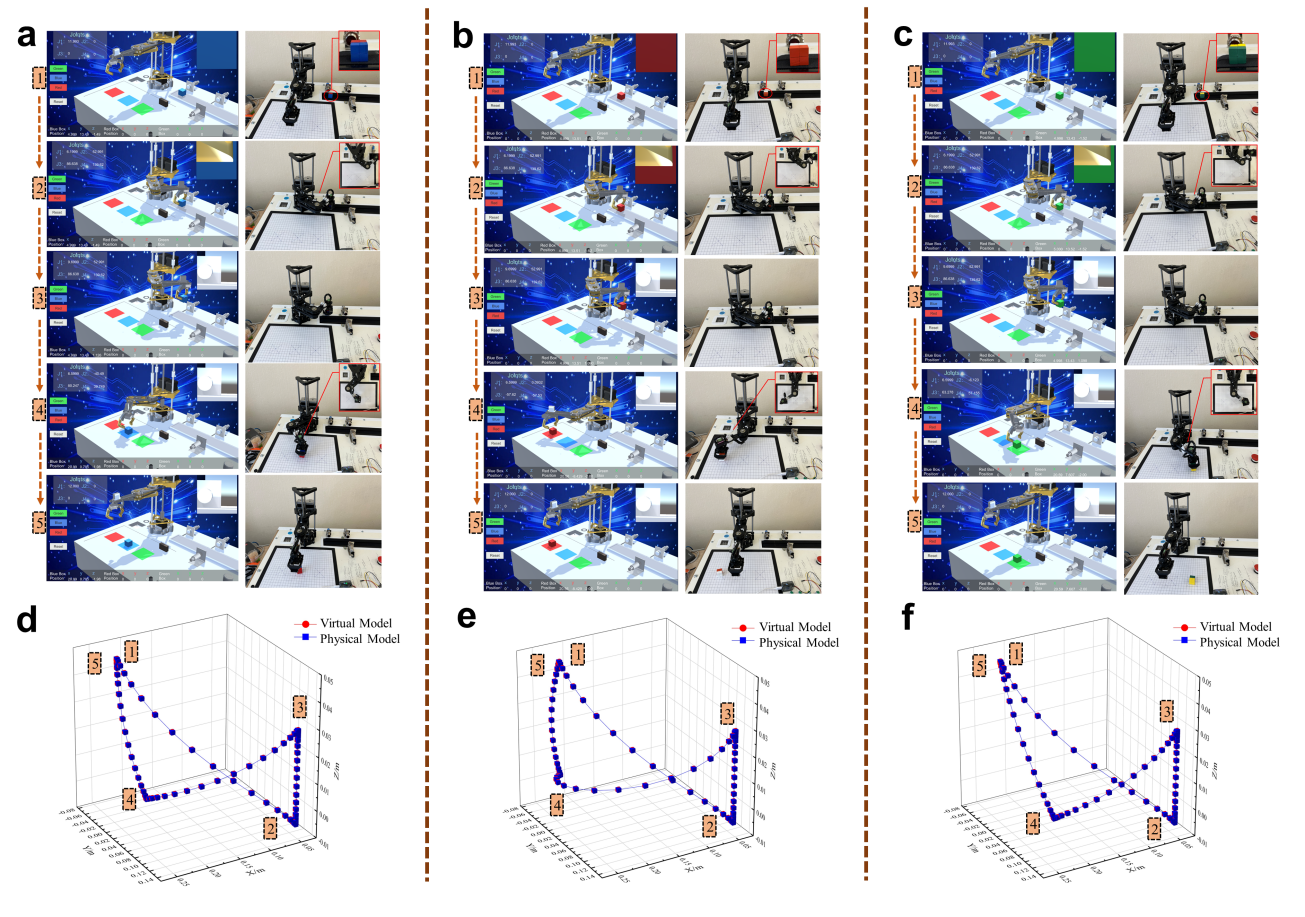
Virtual robot simulation results and physical-robot operational results. (**a**–**c**) The virtual robot simulates the planning motion and loads it into the physical robot in three groups of experiments. (**d**–**f**) Comparison of motion trajectory between virtual-robotic gripper and physical-robotic gripper in three groups of experiments.

Figure 17a–c show the operational results of the virtual simulation system and the real robot. It was observed that, following the operational route planned by the virtual simulation system, the real SCARA robot successfully reached each target point and performed the grasp-and-release actions on the objects at the target points. In three experiments, the physical-robot platform, relying on the color recognition program built by the virtual simulation system, successfully identified the colors of the target objects and completed the color sorting. Figure 17d–f compare the end-effector trajectories of the virtual and physical robots in three experiments. The physical robot’s trajectory in three-dimensional space coincided with the virtual model’s operational results, indicating that the virtual simulation system accurately mapped the physical-robot platform’s operational state. Furthermore, the spatial structure of the virtual system matched the real experimental space. In the virtual system’s simulations, the coordinate data of each target point originated from the virtual space coordinates, but the final computed results were accurately mapped to the physical space. As a digital-twin simulation of the physical-robot platform, the virtual-robot system achieved dual mapping of the robot model and spatial coordinates. It has provided a safer and more convenient virtual experimental platform for testing and controlling robot algorithms.

## 7. Conclusions

This paper addresses the high testing costs of physical-robot algorithms and the complexity of hybrid robot control systems by proposing a method for constructing a digital-twin system for SCARA robots based on modular communication control. The method uses the data-processing layer as the center for data computation and control. By establishing a modular communication system at the functional level, it controls the data collection and transmission between platforms using simple command statements, thereby achieving synchronized virtual and physical movements of the SCARA robot. Experimental results demonstrate that the modular data communication system constructed allows the data-processing layer to use encapsulated control statements to quickly achieve functions such as virtual mapping of the robot, virtual control of the entity, and virtual prediction of the entity. The system also accomplishes cross-platform data communication control, enhances the functionality of offline teaching, and reduces the development costs of robot control systems, thus providing a reference for the development of low-cost robot digital-twin systems.

However, the current system we have constructed lacks intelligent robotic trajectory planning. With the development of deep learning and artificial intelligence, these technologies play an essential role in advancing robotic algorithms and enhancing the intelligence of digital-twin systems. Future research will integrate deep-learning algorithms to incorporate automated trajectory planning for robots within the digital twin system, aiming to improve system functionality and simulation intelligence.

## Figures and Tables

**Table 1:** SCARA robot linkage parameters.

Joint	Joint Angle/°	Link Offset/m	Link Length/m	Link Twist/°
J1	0	$D_{1}$	0.067	0
J2	$\vartheta_{2}$	−0.017	0.092	0
J3	$\vartheta_{3}$	−0.01	0.095	0
J4	$\vartheta_{4}$	−0.04	0	0

## Data Availability

The original contributions presented in the study are included in the article; further inquiries can be directed to the corresponding author.
